# Providing oxygen to children and newborns: a multi-faceted technical and clinical assessment of oxygen access and oxygen use in secondary-level hospitals in southwest Nigeria

**DOI:** 10.1093/inthealth/ihz009

**Published:** 2019-03-21

**Authors:** Ayobami A Bakare, Hamish Graham, Adejumoke I Ayede, David Peel, Olatayo Olatinwo, Oladapo B Oyewole, Kayode R Fowobaje, Shamim Qazi, Rasa Izadnegahdar, Trevor Duke, Adegoke G Falade

**Affiliations:** 1 Department of Paediatrics, University College Hospital, Ibadan, Nigeria; 2 Centre for International Child Health, University of Melbourne, MCRI, Royal Children’s Hospital, Parkville, Australia; 3 Department of Paediatrics, University of Ibadan, Ibadan, Nigeria; 4 Ashdown Consultants, Hartfield, UK; 5 Biomedical Services, University College Hospital, Ibadan, Nigeria; 6 Department of Maternal, Newborn, Child and Adolescent Health, WHO, Nigeria; 7 Bill and Melinda Gates Foundation, Seattle, WA, USA

**Keywords:** children, concentrators, hypoxaemia, newborns, oxygen therapy

## Abstract

**Background:**

Oxygen is an essential medical therapy that is poorly available globally. We evaluated the quality of oxygen therapy in 12 secondary-level Nigerian hospitals, including access to oxygen equipment, equipment functionality, healthcare worker knowledge and appropriateness of use.

**Methods:**

We conducted a three-part evaluation of oxygen access and use involving: (1) facility assessment (including technical evaluation of oxygen equipment), (2) clinical audit (children and neonates admitted January 2014–December 2015) and (3) survey of healthcare worker training and experience on the clinical use of oxygen (November 2015).

**Results:**

Oxygen access for children and newborns is compromised by faulty equipment, lack of pulse oximetry and inadequate care practices. One hospital used pulse oximetry for paediatric care. Eleven hospitals had some access to oxygen supplies. Testing of 57 oxygen concentrators revealed two (3.5%) that were ‘fit for use’. Overall, 14.4% (3708/25 677) of children and neonates received oxygen some time during their admission; 19.4% (1944/10 000) of hypoxaemic children received oxygen; 38.5% (1217/3161) of children who received oxygen therapy were not hypoxaemic.

**Conclusions:**

Oxygen access for children in Nigerian hospitals is poor, and likely results in substantial excess mortality. To improve oxygen access for children globally we must focus on actual provision of oxygen to patients—not simply the presence of oxygen equipment at the facility level. This requires a systematic approach to improve both oxygen (access [including equipment, maintenance and affordability]) and oxygen use (including pulse oximetry, guidelines and continuing education).

## Introduction

Oxygen is an essential medication that is important for treating hypoxaemia from pneumonia, other lung diseases and many other conditions (e.g. sepsis, meningitis, trauma and complications of prematurity) and situations (e.g. emergency, obstetric and peri-operative care).^1^ Global estimates suggest that at least 10–15% of children hospitalized with pneumonia, malaria or meningitis, and about 20% of sick neonates, have hypoxaemia.^2^ Hypoxaemia is a major risk factor for death—increasing the odds of death fivefold among children with acute lower respiratory infection.^3^ Improving the provision of oxygen to children in hospital can reduce inpatient childhood mortality by 10–20%, and pneumonia mortality by 20–40%.^4,5^ However, despite high need, clear evidence of benefit, and global recognition as an essential medicine, oxygen therapy is poorly available for many sick children and adults globally.^1,6^

Providing oxygen to patients requires reliable access to medical oxygen and appropriate oxygen use.^5^ Oxygen access can be limited by lack of equipment, poor equipment functionality or cost.^5^ Good oxygen use requires prompt and accurate recognition of hypoxaemia, correct administration of oxygen therapy and regular monitoring, as well as treatment of the underlying condition. Pulse oximetry, a non-invasive method of measuring blood oxygen levels, is the preferred method to detect hypoxaemia (especially in resource-limited settings where blood gas analysis is rarely feasible),^7,8^ as clinical signs of hypoxaemia are inaccurate.^9,10^ Healthcare workers also need the skills to administer oxygen according to guidelines, and a work environment that ensures they give oxygen to the right patient, at the right time, every time.^5^

Nigeria has a high under-5 y mortality rate (104.3 under-5 y deaths per 1000 live births),^11^ and pneumonia is the number one killer of under-5 y children in Nigeria, responsible for about 140 520 (19%) under-5 y deaths in 2016.^12^ The Nigerian Government has recognized child pneumonia as a priority, and has identified oxygen therapy as an important strategy to reduce deaths from childhood pneumonia.^13,14^

This study aimed to evaluate the quality of oxygen therapy currently available to children and neonates in 12 secondary-level Nigerian hospitals, including: (1) functionality of oxygen source(s); (2) appropriateness of oxygen use; and (3) healthcare workers’ knowledge and experience using oxygen therapy.

## Methods

### Participants

We conducted our study in 12 secondary health facilities in southwest Nigeria, as part of a large field trial to improve oxygen provision to children (UTN U1111-1193-6364).^15^ We selected sites that would be representative of medium-sized hospitals (government or mission) that regularly admit children (described in detail elsewhere).^15^ Our inclusion criteria required hospitals to be secondary health facilities that admitted at least 150 children per year. Our initial focus was on government hospitals in Oyo state. When we could not achieve a sufficient number of hospitals, we decided to include hospitals from other states in southwest Nigeria that met the inclusion criteria. See Appendix 1 (supplementary data) for more detail on participating hospitals.

### Procedures

Our study involved three parts: cross-sectional facility assessment (including technical evaluation of oxygen equipment); retrospective audit of oxygen-related care practices for children and newborns (2 y of data); and assessment of healthcare worker oxygen knowledge and experience.

In April 2015, a team of doctors, technicians and nurses conducted a cross-sectional assessment of each hospital’s capacity to provide oxygen therapy to patients. We used a Facility Assessment Form (adapted from previously used WHO facility assessment tools)^16–18^ to collect data on a broad range of structural, technical and clinical factors that may influence the provision of safe and effective oxygen therapy. The study team collected data during field visits from direct observation and interviews with key informants (including hospital directors, medical officers, nursing officers, technicians, medical records staff and other administrative staff). After completion of the field visit, the study coordinators collated the data and returned it to the medical director (or equivalent) for confirmation of accuracy, and performed follow-up visits and phone calls as necessary. Our technicians followed up the initial facility assessment with a more detailed technical assessment of the existing oxygen concentrators at each hospital, using a standardized data collection tool (Appendix 2) and calibrated oxygen analysers (Longfian Scitech, Baoding China; Cambridge-Sensotec, St Ives, UK). We also recorded informal feedback from technicians and hospital staff during all field visits.

We conducted a retrospective clinical audit of oxygen therapy for children using data extracted from patient case notes. Trained research nurses reviewed ward admission books and administrative records to identify all children (age <15 y) and neonates admitted during the study period (January 2014–December 2015). They extracted data from patients’ case notes using a standardized case report form that had been successfully pilot-tested. We collected demographic and clinical data, including detailed data on oxygen use, diagnoses (using WHO case definitions) and outcomes.

In October and November 2015, we assessed healthcare workers’ knowledge of and experience with the use of oxygen using a standardized written test (Appendix 3). This was administered before and after a basic training module on pulse oximetry, which was conducted at the time of distributing pulse oximeters for the first phase of the main field trial.^15^ The test involved a series of Yes/No questions and a set of scenarios, designed to test basic knowledge and decision-making skills about oxygen therapy, and some questions on prior oxygen-related training.

### Analysis

We present descriptive statistics for health facility characteristics, including the presence of oxygen equipment, associated guidelines and the functionality of oxygen concentrators. We defined a concentrator as being fit for use if: (1) it produces oxygen purity of at least 85%, meeting the WHO recommendation for medical oxygen^8^ and (2) its electrical configuration is appropriate for use in Nigeria.

We report summary statistics to describe the study population, healthcare worker characteristics and the appropriateness of oxygen and pulse oximetry use. We used WHO definitions for hypoxaemia (SpO_2_<90%) and signs of hypoxaemia (any of fast breathing, head nodding, central cyanosis, respiratory rate ≥70 breaths per min, inability to drink due to respiratory distress and grunting with every breath).^8^ We used data from the facility assessment to identify what proportion of oxygen was supplied from functional and non-functional concentrators, and combined this with clinical data to calculate the number of children who received oxygen from a concentrator producing substandard oxygen (<85% purity) (Appendix 4).

We report summary statistics of the healthcare workers who participated in knowledge tests and present descriptive statistics on healthcare worker knowledge, including mean scores with 95% confidence intervals. We report median and interquartile ranges for parameters that are not normally distributed. We used simple linear regression to look for associations between healthcare worker characteristics and experience, and score outcomes. We then used backward step-wise regression to build a multiple linear regression model that included the most relevant variables.

## Results

### Oxygen access

Eleven of the 12 hospitals surveyed had some access to oxygen supplies at the time of assessment (Table 1). Four hospitals used oxygen cylinders alone. Eight hospitals used oxygen concentrators (three hospitals relied mostly on oxygen concentrators and five hospitals used both cylinders and concentrators). Our audit of oxygen practices revealed that staff at 10 of the hospitals had used oxygen for children or neonates during the 2014–2015 study period. Three hospitals had a pulse oximeter on site, but only a single hospital reported routine use of pulse oximeters for children. Our clinical audit confirmed that no other hospitals had been using pulse oximeters prior to our introduction of pulse oximeters in October–November 2015.

**Table 1.** Results from facility assessment of oxygen access at 12 secondary-level hospitals in southwest Nigeria

Hospital identification number	H1	H2	H3	H4	H5	H6	H7	H8	H9	H10	H11	H12
Hospital type	Mission	Mission	State	State	State	Mission	State	State	State	Mission	Mission	State
Paediatric beds	70	32	25	36	60	20	48	46	13	63	14	36
(child+neonatal)	(40+30)	(20+12)	(21+4)	(16+20)	(44+16)	(15+5)	(20+28)	(22+24)	(9+4)	(38+25)	(12+2)	(26+10)
Admissions, January 14–December 15	4530	863	1696	3908	1655	699	5327	1721	284	4087	637	588
Child	2056	772	1555	2028	1357	488	3181	1104	281	2612	602	587
Neonate	2474	91	141	1880	298	211	2146	617	3	1475	35	1
Hospital staffing												
Access to paediatrician	Yes^ii^	No^i^	Yes	Yes	Yes	Yes	Yes	No	No^i^	Yes^ii^	Yes^ii^	No^i^
Doctors—entire hospital	4	4	2	11	17	5	16	12	7	6	6	7
Nurses—child/newborn wards (no. of paediatric-trained)	18	7	16 (2)	33 (3)	62	9 (2)	26	31	11	18	4	26
Hospital oxygen supply												
Oxygen cylinders	Yes^iv^	Yes^iv^	Yes^iv^	Yes	Yes	Yes^iv^	Yes^iii^	Yes	Yes^iv^	Yes	Yes^iv^	Yes^iv^
Oxygen concentrators^v^	Yes^v^	Yes^v^	Yes^v^	Yes^v^	No	Yes	Yes^v^	No	No	Yes^v^	Yes^v^	No
Pulse oximeters^vi^	0	0	0	0	3	1	0	0	0	0	1	0
Oxygen delivery devices												
Nasal prongs	Yes	No	Yes	Yes	Few	Few	Few	Few	Few	Yes	Yes	Few
Nasal catheters	Yes	Yes	Yes	Yes	Yes	Few	No	Yes	No	Few	No	No
Washed and reused	Rarely	No	Yes	Yes	Yes	Yes	No	Yes	No	Yes	Yes	Yes
Oxygen cost (per day)^vii^	N1000	N12000	N1500	N4000	N3500	N9600	FREE	N3600	N7500	N19200	N6000	N2400
US$	$5.43	$65.22	$8.15	$21.74	$19.02	$52.17		$19.57	$40.76	$104.35	$32.61	$13.04

Notes: neonate ≤28 d; child 29 d–15 y; ^i^family medicine consultant; ^ii^part-time; ^iii^piped system connected to large oxygen cylinder; ^iv^not available in paediatric areas; ^v^present but not fit for use (see Table 2 for details); ^vi^H6 was the only hospital routinely practising pulse oximetry; ^vii^average daily cost per patient (individual hospitals variably charged per hour, per day, per patient, per cylinder), with conversion to US$ at 1 January 2015 exchange rate (184:1).

We tested a total of 57 oxygen concentrators across the eight facilities (Table 2). Hospitals had acquired the concentrators through donation from missionary and international agencies and/or through direct procurement from local suppliers. Procurement was usually handled by the hospital head/administrator or (less commonly) the State Ministry of Health. The majority of oxygen concentrators turned on and blew gas (50/57, 87.7%). Of these, most were blowing air (24/50, 48%) or concentrations of oxygen below medical oxygen standards (23/50, 46%). Overall, two (3.5%) concentrators were ‘fit for use’ (producing >85% oxygen and electrically compatible). No concentrators had a clean external filter (a marker of equipment maintenance).

**Table 2.** Results of technical assessment of oxygen concentrators at participating hospitals in southwest Nigeria

Site	Brand (number)	Turns on		O_2_ concentration at 5 LPM flowrate^i^			Fit for use	Clean filter	Median h of use (range)
			Air (20.9%)	22–49%	50–85%	>85%			
**H1**	**n=15**	**13**	**10**	**1**	**2**	**0**	**0**	**0**	**NA**
	G+M (9)	8	7	0	1	0	0	0	
	Nidek (5)*	4	3	0	1	0	0	0	
	Cole (1)	1	0	1	0	0	0	0	
**H2**	**n=9**	**9**	**3**	**0**	**5**	**1**	**0**	**0**	**10 791 (0–25 251)**
	Airsep (6)^ii^,*	6	0	0	5	1	0	0	
	Nellcor (1)	1	1	0	0	0	0	0	
	‘Leaidal’ (2)	2	2	0	0	0	0	0	
**H3**	**n=10**	**8**	**3**	**0**	**5**	**0**	**0**	**0**	**551 (214–3343)**
	Microfield (3)	2	0	0	2	0	0	0	
	Medifield (2)	2	0	0	2	0	0	0	
	Longfei (1)	1	1	0	0	0	0	0	
	MA-Donax (1)	1	1	0	0	0	0	0	
	Unknown (1)	0	0	0	0	0	0	0	
	Unknown (1)	1	0	0	1	0	0	0	
	Laeidal (1)	1	1	0	0	0	0	0	
**H4**	**n=4**	**2**	**1**	**1**	**0**	**0**	**0**	**0**	**6996**
	Longfei (3)	2	1	0	0	0	0	0	
	Laadidal (1)	0	0	1	0	0	0	0	
**H5**	**None**	**N/A**	**N/A**	**N/A**	**N/A**	**N/A**	**N/A**	**N/A**	**N/A**
**H6**	**n=10**	**10**	**4**	**0**	**4**	**2**	**2**	**0**	**11 593 (6996–23 921)**
	CAIRE (10)*	10	4	0	4	2	2	0	
**H7**	**n=2**	**2**	**1**	**1**	**0**	**0**	**0**	**0**	**-**
	Longfei (1)	1	0	1	0	0	0	0	
	Techmel (1)	1	1	0	0	0	0	0	
**H8**	**None**	**N/A**	**N/A**	**N/A**	**N/A**	**N/A**	**N/A**	**N/A**	**N/A**
**H9**	**None**	**N/A**	**N/A**	**N/A**	**N/A**	**N/A**	**N/A**	**N/A**	**N/A**
**H10**	**n=2**	**2**	**1**	**0**	**1**	**0**	**0**	**0**	**-**
	Longfei (1)	1	1	0	0	0	0	0	
	Nidek (1)*	1	0	0	1	0	0	0	
**H11**	**n=5**	**4**	**1**	**2**	**1**	**0**	**0**	**0**	**25 511**
	Hospibrand (3)	3	1	2	0	0	0	0	
	Oxytime (1)	0	0	0	0	0	0	0	
	Unknown (1)	1	0	0	1	0	0	0	
**H12**	**None**	**N/A**	**N/A**	**NA**	**N/A**	**N/A**	**N/A**	**N/A**	**N/A**
**TOTAL**	**N=57**	**50**	**24**	**5**	**18**	**3**	**2**	**0**	**10 823**
		(87.7%)	(42.1%)	(8.8%)	(31.6%)	(5.3%)	(3.5%)	(0%)	(0–25 511)

Notes: LPM, litres per min; ^i^tested at 5 LPM or specified maximum; ^ii^one working but with electrical incompatibility; *has clearance from the US Food and Drug Administration and/or the Conformité Européenne mark via the Declaration of Conformity (to ISO 8359).

We identified various technical problems, including faulty sieve beds (small cylindrical units inside oxygen concentrators that contain zeolite, which helps to remove nitrogen from the air), leaking humidifiers, faulty oxygen concentrator knob, no low-oxygen alarm, electrical faults and lack of routine preventive maintenance for oxygen equipment. Users reported that they did not know the concentrators were producing poor-quality oxygen, and expressed dissatisfaction and frustration with the current situation. Hospital staff (nurses, doctors, technicians and administrators) identified varied reasons for oxygen equipment failure (Box 1). Staff showed particular interest in how they could access better quality concentrators. Following our evaluation, hospital staff removed all the concentrators certified ‘not fit for use’ from clinical use.

**Box 1.** Key reasons identified by hospital staff (nurses, doctors, technicians and administrators) for oxygen equipment failure

Procurement Lack of knowledge on equipment selection. Purchase of refurbished and low-quality oxygen concentrators. Donation of poor-quality oxygen concentrators. Lack of quality control checks on equipment donation for hospitals. Maintenance Lack of knowledge and technical skills on maintenance and repair. Lack of routine equipment maintenance procedures. Non-availability of spare parts and maintenance tools. Poor management support. Lack of training or support for nurses and doctors. Lack of training or support for technicians. Poor planning and inadequate financial support for equipment sustainability. Power Power incompatibility. Power surges damaging equipment.

Eleven of the 12 hospitals required patients to pay for oxygen (and other services and supplies). The median cost per day of oxygen was approximately 3800 Naira (range 0–19 200; US$ 20.65, 0–104.35).

### Oxygen use

During 1 January 2014–31 December 2015, a total of 25 995 children (aged <15 y) were admitted to the 12 participating hospitals. The median age was 9 month, and approximately one-third were neonates (aged <28 d).

Overall, 14.4% (3708/25 677) of children received oxygen therapy at some point during their admission (Table 3a). Pulse oximetry was rarely used (with the exception of H6), with health workers relying on clinical signs to decide when to commence or stop oxygen therapy for patients. Overall, 19.4% (1944/10 000) of children who were hypoxaemic (SpO_2_<90% or signs of hypoxaemia if SpO_2_ not recorded) on admission received oxygen (Table 3b). Hypoxaemic neonates and infants were more likely to be given oxygen than older children. Children with documented SpO_2_<90% were more likely to be given oxygen than those who had signs of hypoxaemia (134/192 69.8% vs 2011/10507 19.1%). Conversely, 61.5% (1944/3161) of children who received oxygen therapy had evidence of hypoxaemia (SpO_2_<90% or signs of hypoxaemia if SpO_2_ not recorded), with negligible variation with age.

**Table 3a.** Pulse oximetry and oxygen use for children <15 y at 12 hospitals in southwest Nigeria (January 2014–December 2015)

	Age group				Overall
	<28 d	28 d–11 months	12–59 months	≥60 months	
Proportion with pulse oximetry documented on admission	407/9372 (4.3%)	254/4959 (5.1%)	470/8563 (5.5%)	171/2783 (6.1%)	1302/25 677 (5.1%)
Proportion with pulse oximetry documented at any time during stay	503/9372 (5.4%)	279/4959 (5.6%)	522/8563 (6.1%)	199/2783 (7.1%)	1503/25 677 (5.9%)
Proportion who received oxygen therapy on admission	1888/9372 (20.2%)	581/4959 (11.7%)	593/8563 (7.0%)	99/2783 (3.6%)	3161/25 677 (12.3%)
Proportion who received oxygen therapy at any time during stay	2134/9372 (22.7%)	703/4959 (14.2%)	725/8563 (8.5%)	146/2783 (5.3%)	3708/25 677 (14.4%)
Proportion with hypoxaemia^1^ on admission	3428/9372 (36.6%)	2135/4959 (43.1%)	3423/8563 (39.9%)	1014/2783 (36.4%)	10 000/25 677 (38.9%)
Proportion with SpO_2_<90% on admission	77/407 (19.0%)^2^	52/254 (20.5%)^2^	46/470 (9.9%)^2^	13/171 (7.6%)^2^	188/1302 (14.4%)^2^
Proportion with SpO_2_<90% at any time	108/503 (21.5%)^2^	65/279 (23.3%)^2^	63/522 (12.1%)^2^	25/199 (12.56%)^2^	261/1503 (17.4%)^2^
Proportion with signs of hypoxaemia^3^ on admission	3518/9372 (37.5%)	2237/4959 (45.1%)	3656/8563 (42.7%)	1096/2783 (39.4%)	10 507/25 677 (40.9%)

^1^Hypoxaemia defined as SpO_2_<90% if SpO_2_ recorded, or any sign of hypoxaemia if SpO_2_ not recorded (severe respiratory distress, central cyanosis, respiratory rate >70 breaths per min, impaired conscious state).

^2^Denominator is total children who had pulse oximetry documented (low rate of pulse oximetry usage at all but one hospital).

^3^Signs of hypoxaemia: severe respiratory distress, central cyanosis, respiratory rate >70 breaths per min, impaired conscious state.

**Table 3b.** Appropriateness of oxygen use for children <15 y at 12 hospitals in southwest Nigeria (January 2014–December 2015)

	Age group				Overall
	<28 d	28 d–11 months	12–59 months	≥60 months	
Proportion with hypoxaemia^1^ on admission who were started on oxygen	1106/3428 (32.3%)	413/2135 (19.3%)	369/3423 (10.8%)	56/1014 (5.5%)	1944/10 000 (19.4%)
Proportion with SpO2<90% on admission who were started on oxygen	62/77 (80.5%)^2^	35/53 (66.0%)^2^	29/49 (59.2%)^2^	8/13 (61.5%)^2^	134/192 (69.8%)^2^
Proportion with signs of hypoxaemia^3^ on admission who were started on oxygen	1136/3518 (32.9%)	423/2237 (18.9%)	385/3656 (10.6%)	67/1096 (6.1%)	2011/10 507 (19.1%)
Proportion of those who received oxygen on admission that had hypoxaemia^1^	1106/1888 (58.58%)	413/581 (71.1%)	369/593 (66.4%)	56/99 (69.7%)	1944/3161 (61.5%)

^1^Hypoxaemia defined as SpO_2_<90% if SpO_2_ recorded, or any sign of hypoxaemia if SpO_2_ not recorded (severe respiratory distress, central cyanosis, respiratory rate >70 breaths per min, impaired conscious state).

^2^Denominator is total children who had pulse oximetry documented (low rate of pulse oximetry usage at all but one hospital).

^3^Signs of hypoxaemia: severe respiratory distress, central cyanosis, respiratory rate >70 breaths per min, impaired conscious state.

Hospital staff reported limited exposure to preservice training on pulse oximetry (approximately 32%) or oxygen use (52%), and pretraining assessment showed low knowledge scores (particularly relating to newborns) (Table 4).

**Table 4.** Results of baseline knowledge and reported experience on pulse oximetry and oxygen therapy among health workers at 10 secondary hospitals in southwest Nigeria^a^

General characteristics	
Total participants	N=249
Median age, y (IQR)	34 (27–42)
Sex, F:M (% female)	174:71 (71%)
Role, nurse:doctor (% nurse)	149:65 (70%)
Median years at hospital (IQR)	5 (2.25–10)
Oxygen-related training experience	
Preservice pulse oximetry training: nurses	48/144 (33.3%)
Preservice pulse oximetry training: doctors	18/65 (27.7%)
Preservice oxygen training: nurses	82/139 (59.0%)
Preservice oxygen training: doctors	23/64 (35.9%)
In-service POx/oxygen training (current hospital): nurses	13/144 (9.0%)
In-service POx/oxygen training (current hospital): doctors	3/65 (4.6%)
In-service POx/oxygen training (anywhere else): nurses	24/142 (16.9%)
In-service POx/oxygen training (anywhere else): doctors	8/65 (12.3%)
Test results	Mean (95% CI)
Total score: max. 40	17.6 (16.7–18.4)
Yes/No questions: max. 20	11.2 (10.7–11.8)
Scenarios^b^: max. 20	6.3 (5.8–6.9)
Sample questions	
Correctly identify that pulse oximeters provide heart rate, SpO_2_ and not blood pressure or respiratory rate^c^	22.1% (16.9–27.3)
Correctly identify that a 2-year-old child with fast breathing and SpO_2_ of 87% should be started on oxygen^b^	67.1% (61.2–72.9)
Correctly identify that a small newborn baby with SpO_2_ 99% on oxygen should have the oxygen flowrate reduced^b^	26.9% (21.3–32.5)

Notes:

^POx^Pulse oximetry. See Appendix 3 for test details.

^a^Two hospitals did not do the baseline knowledge test.

^b^5-option best answer scenario with pulse oximetry result displayed.

^c^Composite from 4 individual true/false questions.

Multiple linear regression analysis showed that doctors scored higher than nurses (+4.1 points, 95% CI 2.7–5.7), and longer duration of service was positively associated with score (+0.08 patients per year, 0.02–0.35) (adjusted for age, sex, professional role and duration of service).

Based on clinical data, observation from research nurses and data from our technical assessment of oxygen concentrators (Appendix 4), we estimated that 43% (1595/3708) of children prescribed oxygen during 2014–2015 had been given substandard oxygen therapy (oxygen purity <85%). Overall, approximately 90% (9037/10 000) of children who had evidence of hypoxaemia did not receive appropriate oxygen therapy. We recorded 1105 deaths in the hypoxaemic cohort. Assuming that appropriate oxygen therapy could have reduced case fatality rates in this cohort by 20–40%, we estimated that lack of appropriate oxygen therapy may have contributed to 220–440 excess deaths over this 2 y period (of which 35–70 related to use of faulty equipment).

## Discussion

This study provides insight into oxygen access and use in 12 secondary hospitals in southwest Nigeria and identifies key opportunities to improve oxygen systems in the country. Previous studies have identified deficiencies in oxygen equipment access,^6,19^ equipment functionality^18,20–23^ or the clinical use of oxygen.^23^

Surveys of healthcare workers in low- and middle-income countries have reported that oxygen and pulse oximeters were available at approximately 75% and 60% of hospitals, respectively, with lower availability in smaller hospitals.^6,19^ Other facility assessments reported lower availability of pulse oximeters (<10%) and identified major issues with non-functional concentrators.^18,20–22^ Few studies have looked at how oxygen is being used at the patient level. Multi-hospital studies in Laos and Malawi reported very low use of pulse oximetry for children with pneumonia (<5%) and the Malawi study found that healthcare workers provided oxygen to approximately 22% of eligible patients.^23,24^ Data from Laos identified cost to patients as a major barrier to appropriate oxygen use.^24^

Our study complements existing data by combining all these elements to provide a comprehensive evaluation of oxygen availability and oxygen use across multiple hospitals and a reasonably long time frame (2 y). Our findings are broadly consistent with previous studies, demonstrating that actual oxygen access is significantly poorer than might be suggested by the oxygen equipment that is available on-site. Despite most hospitals (11/12) having an oxygen source, only 20% of the children who warranted oxygen therapy received it.

Clearly, oxygen access must focus on actual provision of oxygen to patients—not simply the presence of oxygen equipment at the facility level. We identified four key barriers to the effective provision of oxygen to patients: low user knowledge and awareness about oxygen therapy (and pulse oximetry); reliance on clinical signs in the absence of pulse oximetry; lack of reliable oxygen source (e.g. concentrator, cylinder); and high cost to patients.

We were surprised to find how poorly many of the oxygen concentrators being used in hospitals were functioning—only 2 of the 57 concentrators tested were fit for use. These findings expand on previous reports on oxygen concentrator failures,^16,20–22,25,26^ and are particularly relevant given growing interest (and investment) in oxygen concentrators as cost-efficient sources of oxygen in Nigeria and elsewhere.^5,13,27^ Our findings highlight the importance of selecting quality concentrators that can work in hot, humid, dusty conditions and building local maintenance capacity to monitor concentrator function and troubleshoot faults before they become irrepairable.^5,16,20,21,28^ In particular, users need to be able to know whether their oxygen concentrators are providing oxygen at adequate purity using oxygen analysers. While many concentrators offer inbuilt oxygen concentration indicators (OCIs) as an option, none of the concentrators evaluated in our study had OCIs and none of the hospitals had access to oxygen analysers.

The WHO and PATH have recently released technical specifications for oxygen concentrators,^29,30^ and independent assessments have found that most concentrators do not meet these specifications.^31,32^ Given the new oxygen concentrators available on the market,^33^ we need further independent testing to determine suitability for use.

When oxygen therapy is available, it is being poorly used—resulting in no oxygen for many who need it, and unnecessary oxygen use for some who do not need it. Our results complement findings from other studies showing that improved provision of oxygen to patients requires as much attention to oxygen use as it does to oxygen supply.^5^ Healthcare workers need pulse oximetry to make rational decisions about oxygen therapy. Therefore, we must ensure that healthcare workers are adequately equipped, trained and supported to make pulse oximetry part of care routines.

In a user-pay environment, cost to patients may be a major barrier to appropriate oxygen use. Data from other studies suggest that addressing affordability is essential to improving the use of oxygen.^24^

This study has some limitations. It represents a point evaluation of current oxygen systems at the hospitals and does not include any improvement or deterioration in practices postintervention. We assessed the appropriateness of oxygen use based on whether children with likely hypoxaemia on admission received oxygen, and without accounting for whether children were given appropriate flowrates for the full duration of time required (which may result in even lower estimates of appropriate oxygen use). We relied on clinical documentation of pulse oximetry and oxygen practices with a focus on the note written at the time of admission to enable correlation with clinical signs. This provided slightly lower proportions than including data from the whole admission (oxygen use 12.3% vs 14.4%, hypoxaemia prevalence 14.4% vs 17.4%). The retrospective nature of our chart review limited our ability to see what was not documented but also removed the risk of unintentionally biasing the healthcare worker. Our estimates of the number of children that received substandard medical oxygen and excess mortality was performed using facility-level data without knowledge of exactly what oxygen source was used for individual patients.

## Conclusion

Oxygen access for children in the 12 secondary-level hospitals in southwest Nigeria is poor, and likely results in substantial excess mortality. To improve oxygen access for children globally we must focus on actual provision of medical oxygen to patients—not simply the presence of oxygen equipment at the facility level. This requires a systematic approach to both improve oxygen access (including equipment, maintenance and affordability) and oxygen use (including pulse oximetry, guidelines and continuing education).

## Supplementary data

Supplementary data are available at *International Health* online (http://inthealth.oxfordjournals.org).

## Supplementary Material

ihz009_Appendix1-Hospital_Descriptions

ihz009_Appendix2-datacollect

ihz009_Appendix3-test

ihz009_Appendix4-calculations

## References

[1] Duke T, Graham SM, Cherian MN, et al Oxygen is an essential medicine: a call for international action. Int J Tuberc Lung Dis. 2010;14(11):1362–8. 20937173 PMC2975100

[2] Subhi R, Adamson M, Campbell H, et al The prevalence of hypoxaemia among ill children in developing countries: a systematic review. Lancet Infect Dis. 2009;9(4):219–27. 19324294 10.1016/S1473-3099(09)70071-4

[3] Lazzerini M, Sonego M, Pellegrin MC Hypoxaemia as a mortality risk factor in acute lower respiratory infections in children in low and middle-income countries: Systematic review and meta-analysis. PLoS One. 2015;10(9):1–17. 10.1371/journal.pone.0136166 PMC4570717 26372640

[4] Duke T, Wandi F, Jonathan M, et al Improved oxygen systems for childhood pneumonia: a multihospital effectiveness study in Papua New Guinea. Lancet. 2008;372(9646):1328–33. 18708248 10.1016/S0140-6736(08)61164-2

[5] Graham H, Tosif S, Gray A, et al Providing oxygen to children in hospitals: a realist review. Bull World Health Organ. 2017;95:288–302. 28479624 10.2471/BLT.16.186676 PMC5407252

[6] Ginsburg AS, Van cleve WC, Thompson MIW, et al Oxygen and pulse oximetry in childhood pneumonia: A survey of healthcare providers in resource-limited settings. J Trop Pediatr. 2012;58(5):389–93. 22170511 10.1093/tropej/fmr103

[7] Duke T, Subhi R, Peel D, et al Pulse oximetry: technology to reduce child mortality in developing countries. Ann Trop Paediatr. 2009;29:165–75. 19689857 10.1179/027249309X12467994190011

[8] World Health Organization (WHO) Manual on Use of Oxygen Therapy in Children, World Health Organization (WHO). 2016.

[9] Wandi F, Peel D, Duke T Hypoxaemia among children in rural hospitals in Papua New Guinea : epidemiology and resource availability—a study to support a national oxygen programme. Ann Trop Paediatr. 2006;26:277–84. 17132292 10.1179/146532806X152791

[10] Dyke T, Lewis D, Heegaard W, et al Predicting hypoxia in children with acute lower respiratory infection: a study in the highlands of Papua New Guinea. J Trop Pediatr. 1995;41(4):196–201. 7563269 10.1093/tropej/41.4.196

[11] United Nations Inter-Agency Group for Child Mortality Estimation (UN IGME) Levels and Trends in Child Mortality Report; 2017 http://data.unicef.org/resources/levels-trend-child-mortality.

[12] WHO and Maternal and Child Epidemiology Estimation Group *Cause-of-Death-2015*; World Health Organization (WHO), 2015.

[13] Federal Republic of Nigeria *National Strategy for the Scale-up of Medical Oxygen in Health Facilities 2017*–*2022*; 2017 http://www.health.gov.ng/index.php/resources/policy-documents/hospital-services.

[14] Federal Republic of Nigeria *National Policy on Medical Oxygen in Health Facilities*; 2017 http://www.health.gov.ng/index.php/resources/policy-documents/hospital-services.

[15] Graham HR, Ayede AI, Bakare AA, et al Improving oxygen therapy for children and neonates in secondary hospitals in Nigeria: study protocol for a stepped-wedge cluster randomised trial. Trials. 2017;18(1):502. 29078810 10.1186/s13063-017-2241-8 PMC5659007

[16] Matai S, Peel D, Wandi F, et al Implementing an oxygen programme in hospitals in Papua New Guinea. Ann Trop Paediatr. 2008;28:71–8. 18318953 10.1179/146532808X270716

[17] Sa’avu M, Duke T, Matai S Improving paediatric and neonatal care in rural district hospitals in the highlands of Papua New Guinea: a quality improvement approach. Paediatr Int Child Health. 2014;34:75–83. 24621233 10.1179/2046905513Y.0000000081 PMC4153412

[18] Hill SE, Njie O, Sanneh M, et al Oxygen for treatment of severe pneumonia in The Gambia, West Africa: a situational analysis. Int J Tuberc Lung Dis. 2009;13(October 2004):587–93. 19383191

[19] Ginsburg AS, Gerth-guyette E, Mollis B, et al Oxygen and pulse oximetry in childhood pneumonia: surveys of clinicians and student clinicians in Cambodia. Trop Med Int Health. 2014;19(5):537–44. 24628874 10.1111/tmi.12291

[20] Bradley BD, Light JD, Ebonyi AO, et al Implementation and 8-year follow-up of an uninterrupted oxygen supply system in a hospital in The Gambia. Int J Tuberc Lung Dis. 2016;20(8):1130–4. 27393551 10.5588/ijtld.15.0889 PMC4937752

[21] Bradley BD, Chow S, Nyassi E, et al A retrospective analysis of oxygen concentrator maintenance needs and costs in a low-resource setting: experience from The Gambia. Health Technol (Berl). 2015;4(4):319–28.

[22] Howie SRC, Hill SE, Peel D, et al Beyond good intentions: lessons on equipment donation from an African hospital. Bull World Health Organ. 2008;86(1):52–7. 18235890 10.2471/BLT.07.042994 PMC2647344

[23] Mccollum ED, Erica B, Preidis GA, et al Multicenter study of hypoxemia prevalence and quality of oxygen treatment for hospitalized Malawian children. Trans R Soc Trop Med Hyg. 2013;107(5):1–13. 23584373 10.1093/trstmh/trt017 PMC4030433

[24] Gray AZ, Morpeth M, Duke T, et al Improved oxygen systems in district hospitals in Lao PDR: a prospective field trial of the impact on outcomes for childhood pneumonia and equipment sustainability. BMJ Paediatr Open. 2017;1(e000083):1–9. 10.1136/bmjpo-2017-000083 PMC5862216 29637121

[25] Gray AZ, Morpeth M Oxygen Therapy Pilot Project, Lao PDR 2011-2013: Bringing affordable and life-saving oxygen to patients in district hospitals (Final Technical Report, September 2014). Centre for International Child Health, University of Melbourne; 2014.

[26] Nabwire J, Namasopo S, Hawkes M Oxygen Availability and Nursing Capacity for Oxygen Therapy in Ugandan Paediatric Wards. J Trop Pediatr. 2018;64(April):97–103. 28486654 10.1093/tropej/fmx033

[27] Ministry of Health Ethiopia National Medical Oxygen and Pulse Oximetry Scale Up Road Map in Ethopia 2016–*2021*. Addis Ababa, Ethiopia: Ministry of Health Ethiopia; 2016.

[28] La Vincente SF, Peel D, Carai S, et al The functioning of oxygen concentrators in resource-limited settings: a situation assessment in two countries. Int J Tuberc Lung Dis. 2011;15(5):693–9. 21756524 10.5588/ijtld.10.0544

[29] World Health Organization Technical Specification for Oxygen Concentrators. Geneva, Switzerland: World Health Organization (WHO); 2015.

[30] PATH Design for reliability : Ideal product requirement specifications for oxygen concentrators for children with hypoxemia in low-resource settings. Seattle, Washington: PATH; 2015.

[31] Peel D, Hons BS, Phil D, et al Oxygen concentrators for use in tropical countries: a survey. J Clin Eng. 2009;34(4):205–9.

[32] Peel D, Neighbour R, Eltringham RJ Evaluation of oxygen concentrators for use in countries with limited resources. Anaesthesia. 2013;68:706–12. 23654218 10.1111/anae.12260

[33] PATH Technology Lansdscape: Oxygen Concentrators. Seattle, Washington: PATH; 2015 http://www.path.org/publications/files/DT_oxygen_concentrators_landscape_tbl.pdf.

