# Supplementary material for: Providing oxygen to children and newborns: a multi-faceted technical and clinical assessment of oxygen access and oxygen use in secondary-level hospitals in southwest Nigeria
Source: Int Health. 2019 Mar 21;12(1):60–8. doi: 10.1093/inthealth/ihz009 (PMC6964224; doi:10.1093/inthealth/ihz009)
Supplement: ihz009_Appendix2-datacollect [file ihz009_appendix2-datacollect.pdf]

APPENDIX 2 – Concentrator assessment form

**Concentrator serial #**	**Concentrator type**		**Hospital**	**Location**	**Age**	**Hours of use**	**Oxygen concentration**		**Oxygen analyser serial #**	**Comments**
	Make	Model					2LPM	5LPM		
