# Supplementary material for: Providing oxygen to children and newborns: a multi-faceted technical and clinical assessment of oxygen access and oxygen use in secondary-level hospitals in southwest Nigeria
Source: Int Health. 2019 Mar 21;12(1):60–8. doi: 10.1093/inthealth/ihz009 (PMC6964224; doi:10.1093/inthealth/ihz009)
Supplement: ihz009_Appendix3-test [file ihz009_appendix3-test.docx]

| **INSTRUCTIONS**  *Test 1* is designed to test the knowledge and skills of doctors and nurses regarding the clinical use of pulse oximetry and oxygen. Individual results must not be made public (without the subject’s consent).   - Please use a black or blue ballpoint pen. - Print all written entries with BLOCK CAPITAL LETTERS. - Mark boxes with a cross (X) where requested (e.g. ). - Answer every question. - If you make an error - draw a line through the error and write the correct value next to it. Date and initial the correction. |
| --- |

| **PART A – General information** | | | | |
| --- | --- | --- | --- | --- |
| **1** | Hospital Name |  | | |
| **2** | Date of birth (dd/mm/yyyy) | __ __ /__ __ /__ __ __ __ | | |
| **3** | Age | __ __ Years | | |
| **4** | Sex | _1_ Male _2_ Female | | |
| **5** | Job Title |  | |  |
| **6** | Job Category | _1_ Nurse _2_ Nurse Assistant _3_ Student Nurse (training) _4_ House Officer / Corps Member _5_ Resident (Family Medicine or Paediatric training)  _6_ Consultant Family Physician _7_ Consultant Paediatrician _8_ Other Doctor (specify):_____________________  _9_ Other Job Category (specify): _______________ | | |
| **7** | How long have you worked at this hospital? | __ __ Years __ __ Months | | |
| **8** | How long have you worked in Paediatrics? | __ __ Years __ __ Months | | |
| **9** | How long have you worked in Maternity or Newborn care? | __ __ Years __ __ Months | | |
| **PART B - Training on pulse oximetry and the use of oxygen for children** | | | | |
| **10** | Did you receive training on pulse oximetry during your Doctor/Nurse training? | | _0_ NO _1_ YES | |
| **11** | Did you receive training on the use of Oxygen for Children during your Doctor/Nurse training? | | _0_ NO _1_ YES | |
| **12** | Have you used the “Pulse oximetry Training Video”?  (provided by the Oxygen Project Team) | | _0_ NO _1_ YES – I used it in the past month _2_ YES – I used it in the past year _3_ YES – I used in more than a year ago | |
| **13** | Have you attended the “Clinical Use of Oxygen” course? (provided by the Oxygen Project Team) | | _0_ NO _1_ YES – I attended in the past month _2_ YES – I attended in the past year  _3_ YES – I attended more than a year ago | |
| **14** | Have you had any other training on Pulse Oximetry or the use of Oxygen for Children at your hospital? | | _0_ NO _1_ YES – I attended in the past month _2_ YES – I attended in the past year  _3_ YES – I attended more than a year ago  *Specify:* | |
| **15** | Have you had any other training on Pulse Oximetry or the use of Oxygen for Children anywhere else? | | _0_ NO _1_ YES – I attended in the past month _2_ YES – I attended in the past year  _3_ YES – I attended more than a year ago  *Specify:* | |

| **PART C – Quiz.** Answer True or False for every question. | | | |
| --- | --- | --- | --- |
|  | EXAMPLE. Nigeria is: | | |
|  | a. | A country in Africa | _1_ TRUE _2_ FALSE |
|  | b. | An island in Asia | _1_ TRUE _2_ FALSE |
| **1** | The **pulse** **oximeter** **measures**: | | |
|  | a. | The patient’s respiratory rate (RR) | _1_ TRUE _2_ FALSE |
|  | b. | The patient’s heart rate (HR) | _1_ TRUE _2_ FALSE |
|  | c. | Percentage of haemoglobin saturated with oxygen | _1_ TRUE _2_ FALSE |
|  | d. | The patient’s blood pressure (BP) | _1_ TRUE _2_ FALSE |
| **2** | **False readings on an oximeter** can be caused by: | | |
|  | a. | Strong lights shining on the probe | _1_ TRUE _2_ FALSE |
|  | b. | Dark skinned patients | _1_ TRUE _2_ FALSE |
|  | c. | Breathing oxygen from a mask | _1_ TRUE _2_ FALSE |
|  | d. | Nail varnish (paint on nails) | _1_ TRUE _2_ FALSE |
| **3** | Regarding **oxygen saturations** (SpO2): | | |
|  | a. | Saturation should be measured in every child that is admitted to hospital | _1_ TRUE _2_ FALSE |
|  | b. | Saturation of 88% is OK in a healthy 2-year-old | _1_ TRUE _2_ FALSE |
|  | c. | Saturation is normally less than 95% in children | _1_ TRUE _2_ FALSE |
|  | d. | Pre-term neonates normally have lower saturations than older children | _1_ TRUE _2_ FALSE |
| **4** | The following clinical signs indicate the **need for** **urgent resuscitation**: | | |
|  | a. | Fever | _1_ TRUE _2_ FALSE |
|  | b. | Severe respiratory distress (e.g. grunting, gasping, severe chest indrawing) | _1_ TRUE _2_ FALSE |
|  | c. | Convulsions (seizures, fits) | _1_ TRUE _2_ FALSE |
|  | d. | Coma (unconscious or barely conscious) | _1_ TRUE _2_ FALSE |
| **5** | **Oxygen concentrators**: | | |
|  | a. | Take a few minutes to reach adequate oxygen concentration | _1_ TRUE _2_ FALSE |
|  | b. | Must be turned off for at least one hour every day | _1_ TRUE _2_ FALSE |
|  | c. | Must have the external filter cleaned every week | _1_ TRUE _2_ FALSE |
|  | d. | Can be used for more than one patient simultaneously | _1_ TRUE _2_ FALSE |
| **6** | To **test whether oxygen is reaching the patient** you should: | | |
|  | a. | Listen for a noise – any noise is good | _1_ TRUE _2_ FALSE |
|  | b. | Look at the flow meter – it shows what flow is reaching the patient | _1_ TRUE _2_ FALSE |
|  | d. | Put the nasal prongs in water and look for bubbles – bubbles show gas flow through the prongs | _1_ TRUE _2_ FALSE |
|  | e. | Look at the colour of the patient’s skin – if they are not blue the oxygen must be working | _1_ TRUE _2_ FALSE |

| **PART D – Scenarios.** Choose the best action for each of the following scenarios. | | |
| --- | --- | --- |
|  | EXAMPLE  **SpO2(%) PR(bpm) 99 117 Pleth** 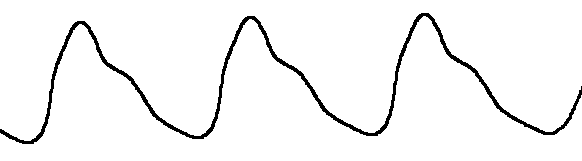 | EXAMPLE: A 4-year-old child has a cough and fever.  _1_ Continue routine care, this is a normal reading  _2_ Start oxygen, the SpO2 is low |
|  |  | _3_ Check again in 10 minutes |
|  |  | _4_ Check the probe placement and try again |
|  |  | _5_ Call for urgent help, this is an Emergency |
| **7** | **SpO2(%) PR(bpm) 97 107 Pleth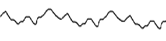** | A 9-month baby is having a convulsion (fit).  _1_ Continue routine care, this is a normal reading |
|  |  | _2_ Start oxygen, the SpO2 is low |
|  |  | _3_ Check again in 10 minutes |
|  |  | _4_ Check the probe placement and try again |
|  |  | _5_ Call for urgent help, this is an Emergency |
| **8** | **SpO2(%) PR(bpm) 87 142 Pleth**  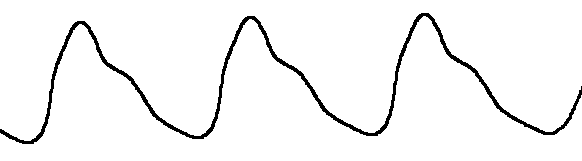 | A 2-year-old child comes to hospital with fast breathing.  _1_ Continue routine care, this is a normal reading |
|  |  | _2_ Start oxygen, the SpO2 is low |
|  |  | _3_ Check again in 10 minutes |
|  |  | _4_ Check the probe placement and try again |
|  |  | _5_ Call for urgent help, this is an Emergency |
| **9** | **SpO2(%) PR(bpm) 96 110 Pleth**  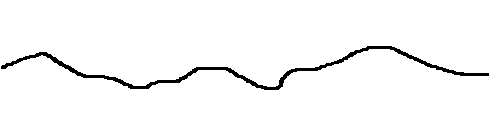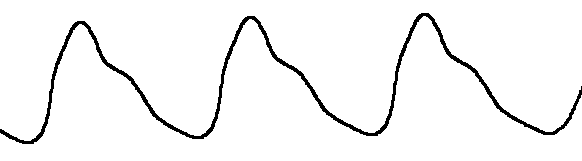 | A 12-month child has a cough and fever.  _1_ Continue routine care, this is a normal reading |
|  |  | _2_ Start oxygen, the SpO2 is low |
|  |  | _3_ Stop oxygen, check again in 10 minutes |
|  |  | _4_ Check the probe placement and try again |
|  |  | _5_ Call for urgent help, this is an Emergency |
| **10** | **SpO2(%) PR(bpm) 99 144 Pleth**  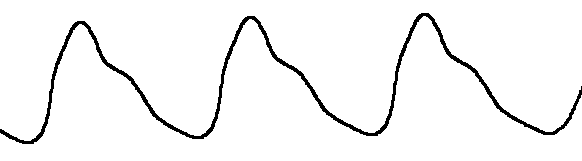 | An 1800g newborn baby is on oxygen for fast breathing.  _1_ Continue current oxygen, this is a normal reading |
|  |  | _2_ Reduce oxygen flow rate, the SpO2 is too high |
|  |  | _3_ Stop oxygen, check again in 10 minutes |
|  |  | _4_ Check the probe placement and try again |
|  |  | _5_ Call for urgent help, this is an Emergency |
