# Supplementary material for: Providing oxygen to children and newborns: a multi-faceted technical and clinical assessment of oxygen access and oxygen use in secondary-level hospitals in southwest Nigeria
Source: Int Health. 2019 Mar 21;12(1):60–8. doi: 10.1093/inthealth/ihz009 (PMC6964224; doi:10.1093/inthealth/ihz009)
Supplement: ihz009_Appendix4-calculations [file ihz009_appendix4-calculations.docx]

| Hospitals | H1 | H2 | H3 | H4 | H5^i^ | H6 | H7 | H8 ^i^ | H9 ^i^ | H10 | H11 | H12 ^i^ | Total |
| --- | --- | --- | --- | --- | --- | --- | --- | --- | --- | --- | --- | --- | --- |
| Children |  |  |  |  |  |  |  |  |  |  |  |  |  |
| Oxygen therapy at any time | 227 | 74 | 207 | 187 | 55 | 96 | 252 | 165 | 0 | 224 | 83 | 4 | 1574 |
| Estimated proportion^ii^ | 1 | 0.2 | 1 | 0.1 | 0 | 0.9 | 0.1 | 0 | 0 | 0.2 | 0.25 | 0 | NA |
| Substandard oxygen therapy^iii^ | 227 | 15 | 207 | 19 | 0 | 86 | 25 | 0 | 0 | 45 | 21 | 0 | 645 |
| Neonates |  |  |  |  |  |  |  |  |  |  |  |  |  |
| Oxygen therapy at any time | 660 | 25 | 41 | 301 | 44 | 94 | 498 | 159 | 0 | 306 | 6 | 0 | 2134 |
| Estimated proportion^ii^ | 1 | 0.2 | 1 | 0.1 | 0 | 0.9 | 0.1 | 0 | 0 | 0.25 | 0.25 | 0 | NA |
| Substandard oxygen therapy^iii^ | 660 | 5 | 41 | 30 | 0 | 85 | 50 | 0 | 0 | 77 | 2 | 0 | 950 |
| TOTAL |  |  |  |  |  |  |  |  |  |  |  |  |  |
| Oxygen therapy at any time |  |  |  |  |  |  |  |  |  |  |  |  | 3708 |
| Substandard oxygen therapy |  |  |  |  |  |  |  |  |  |  |  |  | 1595 |

Notes:

1. Hospital did not have oxygen concentrators
2. Proportion of patients estimated to have received oxygen therapy via oxygen concentrators. Estimate is based on feedback obtained from hospital staff and administrator during baseline assessment visits and technical evaluation of hospitals’ existing oxygen concentrator
3. Substandard oxygen therapy: oxygen purity < 85%

NA Not Applicable
